# Tree Root-Associated Microbial Communities Depend on Various Floor Management Systems in an Intensive Apple (*Malus* × *domestica* Borkh.) Orchard

**DOI:** 10.3390/ijms24129898

**Published:** 2023-06-08

**Authors:** Kamila Łucja Bokszczanin, Sebastian Przybyłko, Karolina Molska-Kawulok, Dariusz Wrona

**Affiliations:** Department of Pomology and Horticulture Economics, Institute of Horticultural Sciences SGGW, Nowoursynowska 159 Str., 02-787 Warsaw, Poland; sebastian_przybylko@sggw.edu.pl (S.P.); karolina_molska@sggw.edu.pl (K.M.-K.); dariusz_wrona@sggw.edu.pl (D.W.)

**Keywords:** microbiome, organic floor management system, rhizosphere, soil organic matter

## Abstract

Regenerative 3agriculture prioritizes soil health to build up organic soil carbon and nitrogen stocks while supporting the active and diverse soil biota that is a prerequisite for maintaining crop productivity and quality in sustainable food production. This study aimed at unravelling the impact of organic and inorganic soil maintenance systems in a ‘Red Jonaprince’ apple (*Malus* × *domestica* Borkh.) orchard on soil microbiota biodiversity and soil physico-chemical properties. During our study, we compared seven floor management systems in terms of microbial community diversity. Fungal and bacterial communities on all taxonomic levels differed largely between systems that augmented organic matter (organic) and other tested inorganic regimes. The dominant phylum of the soil in all management systems was *Ascomycota*. The operational taxonomic units (OTUs) within the *Ascomycota* were largely identified as members of *Sordariomycetes*, followed by *Agaricomycetes*, and both dominated in organic systems versus inorganic. The most prominent phyla, *Proteobacteria*, accounted for 43% of all assigned bacteria OTUs. *Gammaproteobacteria*, *Bacteroidia*, and *Alphaproteobacteria* were predominant in organic samples, while *Acidobacteriae*, *Verrucomicrobiae*, and *Gemmatimonadetes* were more abundant in inorganic mulches.

## 1. Introduction

Soils and crops in orchard agrosystems are particularly vulnerable to climate change and environmental stresses. Decades of intensive agriculture, along with mineral fertilization, have diminished soil organic matter (SOM) content, thereby reducing the fertility and biodiversity of arable lands [1,2]. Consequently, important soil ecosystem services such as nutrient cycling, water regulation, carbon (C) storage, and functional biodiversity are in many cases impaired [2,3]. In these terms, sustainable soil use, aimed at increasing SOM and the occurrence of beneficial microorganisms that act as biofertilizers and biopesticides, plays a crucial role. An active and diverse biota is a prerequisite for maintaining crop productivity and quality, and preservation of these traits is a major goal of sustainable farming [3].

Soil pH and organic matter (OM) have been distinguished as key determinants of rhizosphere microbial communities and can vary in apple (*Malus* × *domestica* Borkh.) orchard soils [4,5]. The microbiome has a decisive impact on soil health, supporting organic matter decomposition, nutrient cycling and buffering, soil structure, redox balance, and the degradation of pollutants. Moreover, it has an impact on plant health and growth through the positive benefits of mycorrhization, symbiotic interaction, and resistance induction, or negatively through pathogenic infection. In the research conducted by Berdeni et al. [6], the arbuscular mycorrhizal fungi (AMF) inoculation significantly increased resistance to *Neonectria ditissima*, a global economically significant fungal pathogen of apple orchards. Our previous research showed that AMF and plant-growth-promoting rhizobacteria (PGPR) significantly increased the concentration of nitrogen (N), phosphorus (P), and potassium (K) in leaves, depending on the N dosage. Symbiosis positively conditioned the K in fruits under a specific N regime [7].

A large proportion of European Union soil is currently subject to unsustainable management practices and, thus, faces a number of challenges, including erosion, degradation, and desertification, as well as a decline in SOM and a loss of biodiversity [8]. Among the key priorities that support the sustainable growth of food production and greener farm practices through eco-schemes, and which are particularly relevant to soils, are increasing the SOM, limitation of nitrogen mineral fertilization, organic farming, crop rotation, and preservation of carbon-rich soil [8]. Thus, sustainable practices are fundamental to the improvement of soil fertility and quality in apple orchards. For instance, mulching materials protect the soil from wind and water erosion and reduce the compaction of soil, which can adversely affect the roots of crops, consequently reducing the growth and development of the plants. Organic mulches increase the content of SOM, prevent water evaporation, limit the growth of weeds, and alter soil temperatures in comparison with bare soil by reducing the soil temperature in summer and raising it in winter. The buffering effect for the temperature on the covered soil also remains in the deeper layers [9,10,11,12]. Cover crop-derived mulches can increase earthworm abundance and mass, as well as the decomposition of disease-harboring leaf litter and burial, potentially reducing the risk of apple scab disease (*Venturia inaequalis*) [13]. The application of organic mulches is more beneficial because these can be decomposed in an appropriate environment, providing nutrients [14]. It has been shown that the use of *Miscanthus* litter contributes to the increase in tree growth and affects the regularity of fruit-bearing [15]. The increment of the trunk cross-sectional area was higher, and fruits were significantly larger for *Miscanthus* sp.-mulched trees when compared to mechanical fallow, herbicide strips, and black polypropylene cover plots [15].

Moreover, microecological imbalance in apple trees’ rhizospheres caused by variation in the soil microbial community is considered the primary cause of apple replant disease (ARD), assisted by increased populations of typical pathogenic fungi *Verticillium* and bacteria *Xanthomonadaceae* and decreased populations of the beneficial bacterial populations *Pseudomonas* and *Bacillus* [16].

The main goal of the present study was assessing the long-term effects of different floor management systems on the diversity of microorganisms in orchard soil, with a special concern to the genetic composition of the rhizosphere microbiome.

## 2. Results

The experiment revealed that the floor management system significantly affected the orchard soil parameters (Table 1). The soil samples taken from the plots mulched with mushroom compost mixed with the soil (FMM) showed higher pH values compared to those of the herbicide strips (HSs), mechanical cultivation (MC), synthetic mulch (BC), *Miscanthus* mulch (MM), and *Miscanthus* mulch mixed with the soil (MMM) combinations. The mulches covering the soil surface also affected the mineral concentration in the soil. Higher phosphorus contents were noted for the FMM combination compared with other treatments. In terms of the potassium content, a very clear relationship was observed. The use of spent mushroom substrate, regardless of the variant used, increased the potassium concentration in the soil in relation to the other combinations. The FMM treatment had a significant effect on the magnesium content. This treatment enriched the soil with magnesium when compared with all of the other combinations except FM. The spent mushroom substrate elevated the soil conductivity. There was an eightfold increase observed for this parameter in the FM and FMM soil samples, as well as higher organic matter content when compared to other tested combinations (Table 1).

### 2.1. High-Throughput Amplicon Sequencing

**Figure 1:**
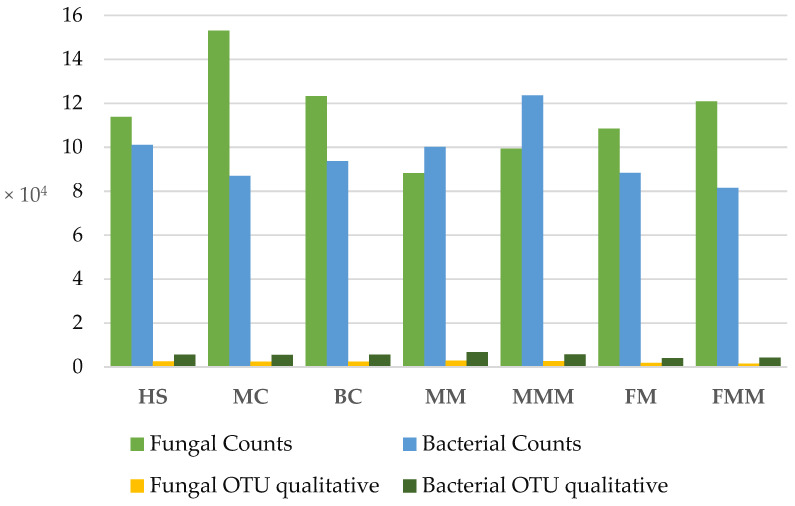
Fungal and bacterial counts and qualitative OTUs of high-throughput amplicon sequencing. Abbreviations: HS—herbicide strip, MC—mechanical cultivation, BC—synthetic mulch, MM—*Miscanthus* mulch, MMM—*Miscanthus* mulch mixed with the soil, FM—mushroom compost, FMM—mushroom compost mixed with the soil.

After paired-end alignments, quality filtering and deletion of chimeric, singletons, and mitochondrial and chloroplast sequences, bacterial 16S rRNA and fungal internal transcribed spacer (ITS) sequences were assigned to 675,350 bacterial and 807,042 fungal operational taxonomic units (OTUs) (Figure 1). The highest ratio of fungal to bacterial sequences was observed in MC (64% of fungal sequences) and the lowest in the MMM sample (44% of fungal sequences). The number of unique observations per sample were 16,301 and 37,580 for bacterial and fungal OTUs, respectively (Figure 1).

**Figure 2:**
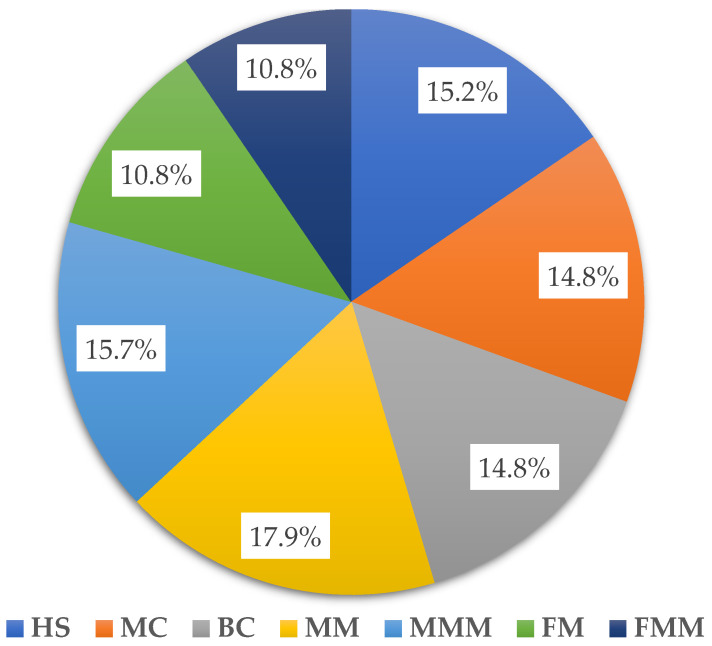
Proportion of total OTUs revealed for tested samples depending on the floor management system in the orchard. Abbreviations: HS—herbicide strip, MC—mechanical cultivation, BC—synthetic mulch, MM—*Miscanthus* mulch, MMM—*Miscanthus* mulch mixed with the soil, FM—mushroom compost, FMM—mushroom compost mixed with the soil.

Relatively low proportions of the total OTUs were assigned in samples collected from plots where spent mushroom substrate was used for mulching (Figure 2). FM and FMM combinations were both characterized by 10.8% of the total OTUs noted in the experiment, respectively, while for other tested floor management systems the average shares ranged from 14.8 to 17.9% (Figure 2).

### 2.2. The α-Diversity Analysis

The Chao, ACE, Shannon, and Simpson indices calculated from the fungal OTUs of all the samples indicated that the α diversity of the FM and FMM samples was lower than that of the other organic and inorganic management systems. Shannon, Chao1, and ACE indices especially suggested that there was greater fungal diversity in the MM and MMM soil systems, while there were localized reductions in the soil microbial diversity in the FM and FMM samples. Similarly, Shannon, Chao1, and ACE indices calculated from the bacterial OTUs showed the highest richness in *Miscanthus*-mulched samples and were reduced in the FM and FMM regimes (Table 2).

### 2.3. Taxonomic Classification

#### 2.3.1. Fungi

**Figure 3:**
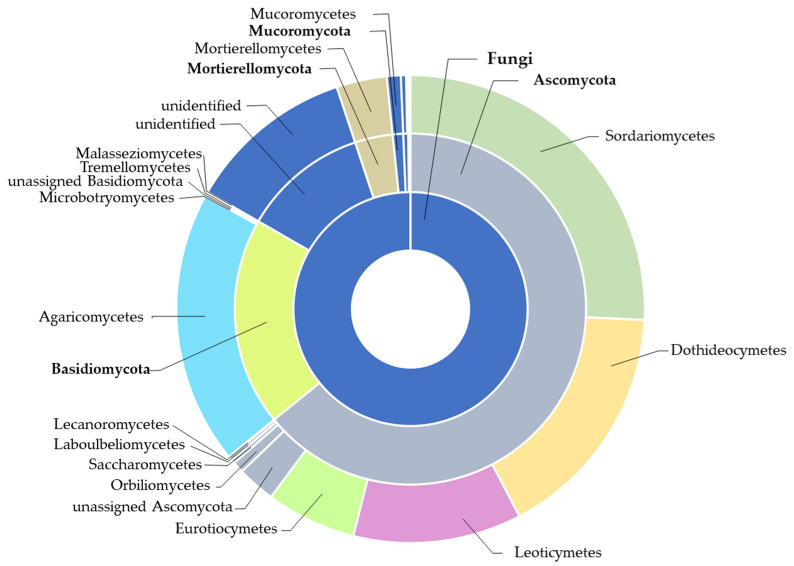
Proportions of fungal phyla and classes of the whole data set based on OTUs [%].

Referencing the representative sequences of all 278,926 fungal OTUs against the database led to their attribution into 12 phyla, 27 classes, 70 orders, 158 families, and 401 species. Among the 12 phyla, the most abundant were *Ascomycota* and *Basidiomycota*, accounting for 64% and 19% of the OTUs, respectively (Figure 3). A total of 11% of the sequences were assigned to ‘unidentified’ (Figure 3).

**Figure 4:**
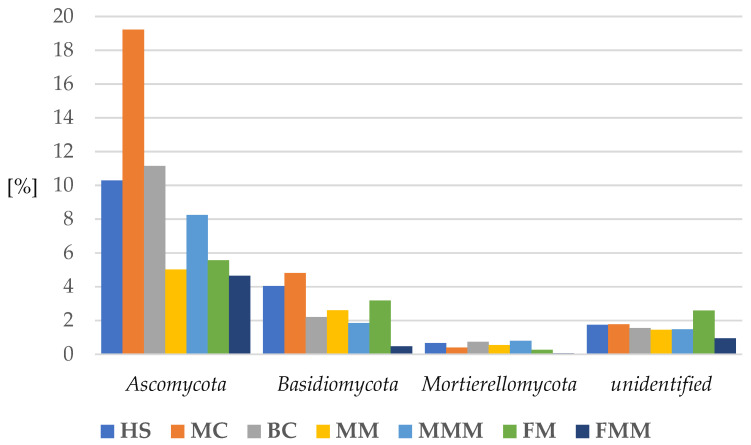
Percentage of the most abundant fungal phyla in apple rhizosphere depending on the soil management system. (HS—herbicide strip, MC—mechanical cultivation, BC—synthetic mulch, MM—*Miscanthus* mulch, MMM—*Miscanthus* mulch mixed with the soil, FM—mushroom compost, FMM—mushroom compost mixed with the soil).

In order to further investigate the diversity of fungal communities, the proportions of the phyla and classes were examined according to the seven soil maintenance systems. Organic managements (MM, MMM, FM, and FMM) showed a slightly reduced proportion of *Ascomycota* (average 6%) when compared to inorganic systems (HS, MC, and BC) (average 10%). Mechanical cultivation revealed the highest proportion of *Ascomycota* (19%). Similarly, *Basidiomycota* were less numerous in organic systems (average 2%) in comparison to inorganic (average 3.5%), with the greatest share in MC (5%) (Figure 4).

**Figure 5:**
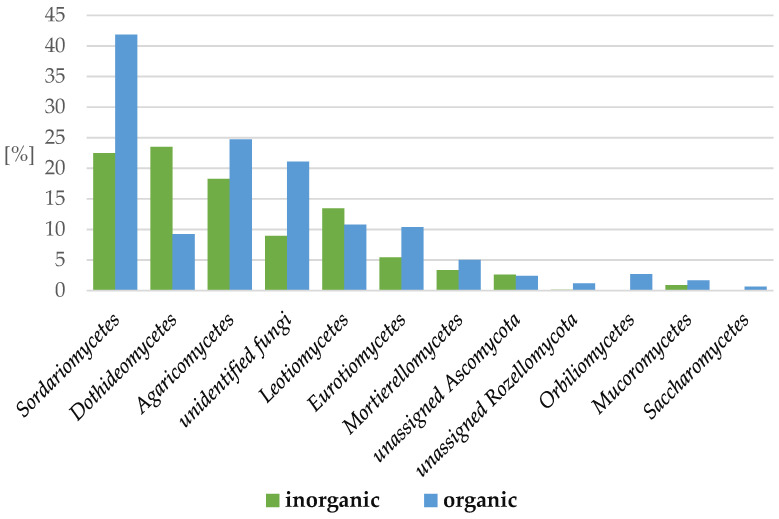
The most abundant fungi classes (more than 1% of OTUs per sample) in apple rhisophere depending on the inorganic or organic soil management system. (inorganic: herbicide strip, mechanical cultivation, synthetic mulch; organic—*Miscanthus* mulch, *Miscanthus* mulch mixed with the soil, mushroom compost, mushroom compost mixed with the soil).

Regarding the class proportions, we distinguished 12 classes represented by more than 1% per sample: *Sordariomycetes* (from 21% in HS to 42% in FMM), *Dothideomycetes* (from 4% in FM to 29% in BC), *Agaricomycetes* (from 7% in FMM to 26% in MM and FM), unidentified fungi (from 6% in MC to 21% in FM), *Leotiomycetes* (from 2% in FM to 16% in MMM), *Eurotiomycetes* (from 3% in BC to 11% in FMM), *Mortierellomycetes* (from 1% in FMM to 6% in MMM), unassigned *Ascomycota* (from 0.6% in HS to 6% in MC), unassigned *Rozellomycota* (2% in FMM), *Orbiliomycetes* (2% in FM), *Mucoromycetes* (1% in MMM), and *Saccharomycetes* (1% in MM). Summarizing, in organic regimes, the following classes prevailed: *Sordariomycetes*, *Agaricomycetes*, unidentified class of *Fungi*, *Eurotiomycetes*, *Mortierellomycetes*, and *Orbiliomycetes*, while *Dothideomycetes* and *Leotiomycetes* predominated in the inorganic samples (Figure 5).

**Figure 6:**
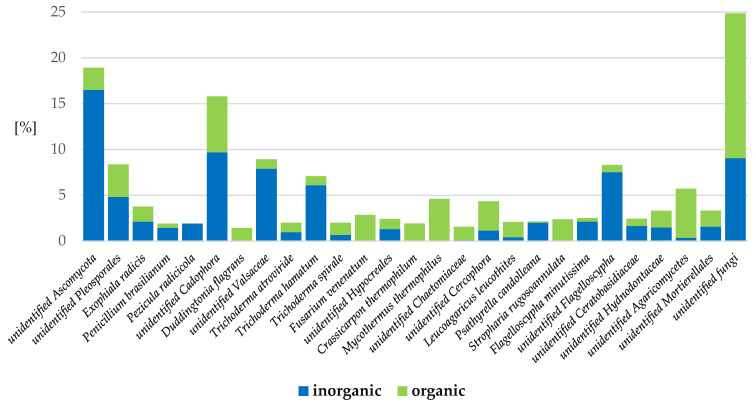
Fungi species in apple rhizosphere with more than 1% abundance, depending on the inorganic or organic soil management system. (inorganic: herbicide strip, mechanical cultivation, synthetic mulch; organic: *Miscanthus* mulch, *Miscanthus* mulch mixed with the soil, mushroom compost, mushroom compost mixed with the soil).

The following species were observed to predominate in organic systems: unidentified fungi (7% more), unidentified species of *Agaricomycetes* (*Basidiomycota*) (5% more), unidentified species of *Cercophora* (*Ascomycota*) (2% more), and *Leucoagaricus leucothites* (*Basidiomycota*) (1% more) (Figure 6).

Fungi species that were exclusively present in organic soil regimes included *Mycothermus thermophilus* (*Basidiomycota*) (5%), *Fusarium venenatum* (*Ascomycota*) (3%), *Stropharia rugosoannulata* (*Basidiomycota*) (2.4%), *Crassicarpon thermophilum* (*Ascomycota*) (1.9%), *Duddingtonia flagrans* (*Ascomycota*) (1%), and unidentified species of *Chaetomiaceae* (*Ascomycota*) (1%) (Figure 6).

In the inorganic systems, more of the following species were identified vs. in the organic systems: unidentified species of *Pteridiospora* (*Ascomycota*) (14% more), unidentified species of *Valsaceae* (*Ascomycota*) (7% more), unidentified species of *Flagelloscypha* (*Basidiomycota*) (7% more), *Trichoderma hamatum* (*Ascomycota*) (5% more), unidentified species of *Cadophora* (*Ascomycota*) (4% more), and unidentified species of *Pleosporales* (*Ascomycota*) (1% more) (Figure 6).

Fungi species that were exclusively present in inorganic soil regimes included *Pezicula radicicola* (*Ascomycota*) (2%) (Figure 6).

#### 2.3.2. Bacteria

**Figure 7:**
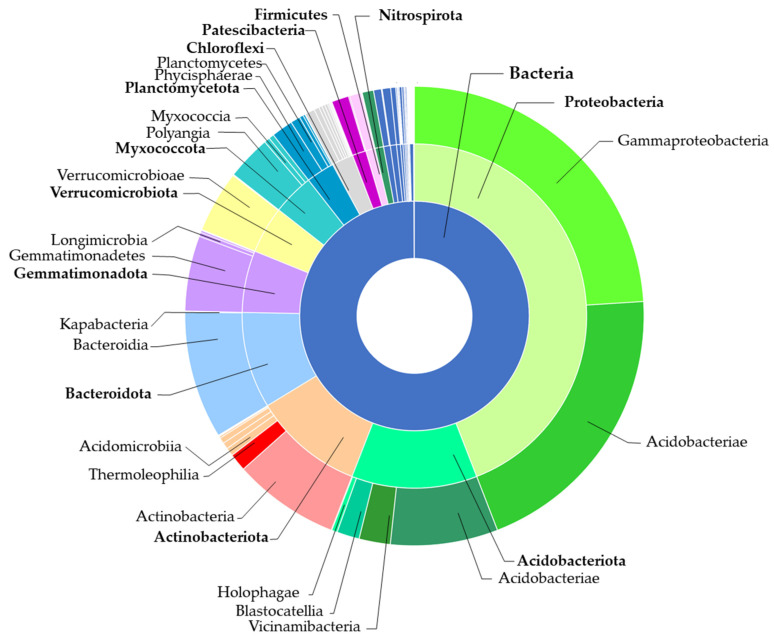
Overview of bacteria phyla and classes of apple rhizosphere microbiomes originated from organic and inorganic soil management systems.

Referencing the representative sequences of all 675,350 bacterial OTUs against database led to their attribution into 33 phyla, 97 classes, 230 orders, 362 families, 767 genera, and 1618 species. Among the phyla, the most abundant were *Proteobacteria*, *Acidobacteriota*, and *Actinobacteriota*, which accounted for 19%, 13.5%, and 11% of all assigned OTUs, respectively (Figure 7).

**Figure 8:**
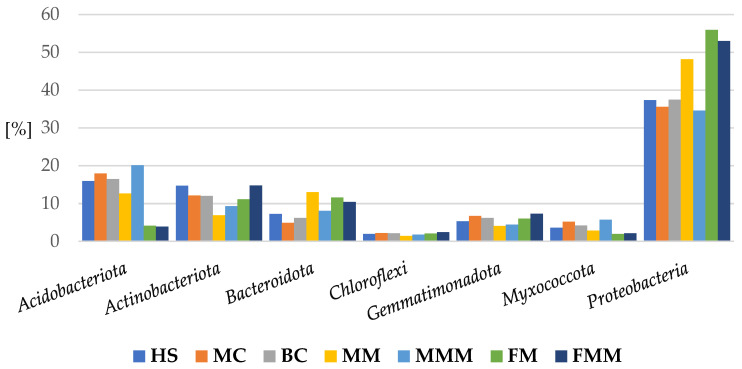
Percentage of most abundant bacteria phyla in apple rhizosphere depending on the soil management system. (HS—herbicide strip, MC—mechanical cultivation, BC—synthetic mulch, MM—*Miscanthus* mulch, MMM—*Miscanthus* mulch mixed with the soil, FM—mushroom compost, FMM—mushroom compost mixed with the soil).

*Proteobacteria* and *Bacteroidota* prevailed in the organic samples vs. inorganic by 11% and 4.6%, respectively. On the contrary, *Acidobacteriota* and *Actinobacteriota* predominated in the inorganic samples vs. organic by 6.5% and 2.4% (Figure 8).

**Figure 9:**
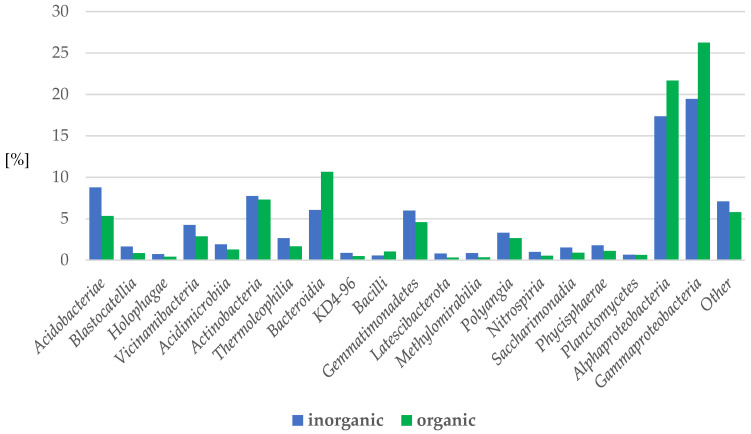
Percentage of bacteria phyla in apple rhizosphere depending on the inorganic or organic soil management system. (inorganic: herbicide strip, mechanical cultivation, synthetic mulch; organic—*Miscanthus* mulch, *Miscanthus* mulch mixed with the soil, mushroom compost, mushroom compost mixed with the soil).

Regarding the class proportions, there were 16 classes represented by more than 1% per sample: *Alphaproteobacteria*, *Acidobacteriae*, *Actinobacteria*, *Bacteroidia*, *Gemmatimonadetes*, *Verrucomicrobiae*, *Vicinamibacteria*, *Thermoleophilia*, *Polyangia*, *Phycisphaerae*, *Saccharimonadia*, *Acidimicrobiia*, *Blastocatellia*, *Nitrospiria*, *Bacilli*, and *Methylomirabilia* (Figure 9).

*Gammaproteobacteria*, *Bacteroidia*, and *Alphaproteobacteria* were predominant in the organic samples versus inorganic by 7%, 5% and 4%, respectively, while *Acidobacteriae*, *Verrucomicrobiae*, and *Gemmatimonadetes* were more abundant in the inorganic mulches than in the organic ones by 3%, 2%, and 1%, respectively (Figure 9).

**Figure 10:**
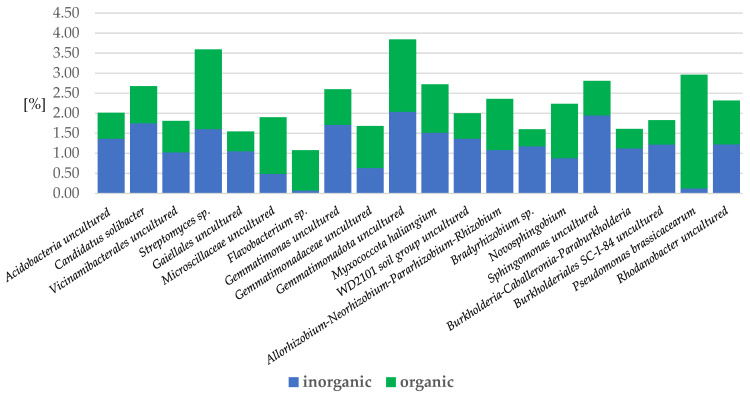
Bacteria species in apple rhizosphere with more than 1% abundance, depending on the inorganic or organic soil management system. (inorganic: herbicide strip, mechanical cultivation, synthetic mulch; organic: *Miscanthus* mulch, *Miscanthus* mulch mixed with the soil, mushroom compost, mushroom compost mixed with the soil).

Analysis of the bacteria species among different soil regimes revealed that in organic samples, *Pseudomonas brassicacearum* (*Proteobacteria*) (2.7% more), uncultured *Microscillaceae* (*Bacteroidota*) bacteria (1% more), *Novosphingobium* sp. (*Proteobacteria*), *Flavobacterium* sp. (*Bacteroidota*) (1% more), *Gemmatimonadaceae* sp. (*Gemmatimonadota*), and *Streptomyces* sp. (*Actinobacteriota*) prevailed (Figure 10).

In inorganic samples, the following species predominated: uncultured *Sphingomonas* bacteria (*Proteobacteria*) (1% more), uncultured bacteria (*Acidobacteriota*) (0.8% more), uncultured *Gemmatimonas* bacteria (*Gemmatimonadota*) (0.8% more), uncultured WD2101 soil group bacteria (*Planctomycetota*) (0.7% more), uncultured *Acidobacteria* (*Acidobacteriota*) (0.7% more), uncultured *Proteobacteri* SC-I-84 bacteria (0.6% more), *Bradyrhizobium* sp. (*Proteobacteria*) (0.7% more), *Burkholderia*-*Caballeronia*-*Paraburkholderia* sp. (*Proteobacteria*) (0.6% more), and uncultured *Thermoleophilia* bacteria (*Actinobacteriota*) (0.5% more) (Figure 10).

## 3. Discussion

It has previously been well-documented that the floor management system affects the most important soil properties that decide its fertility [17,18,19,20,21]. The results published by Przybyłko et al. [22] indicate that the dynamics and direction of changes that occur depend on the soil maintenance used. During three consecutive seasons of different floor management systems, use of the spent mushroom substrate led to an increase in the P, K, and Mg concentrations, as well as in the pH value. On the other hand, Miscanthus straw showed to be a much poorer source of the analyzed macronutrients, although relatively high concentrations of P and K were noted in comparison to the systems tested where no organic litter was used at all [22]. The variations including increasing soil salinity and organic matter content after the spent mushroom application that we observed encouraged us to check to what extent the diversity of tree root-associated microbial communities was affected in such conditions.

The fungal community compositions of soil ecosystems in apple orchards are usually dominated by *Ascomycota* [3,23,24]. Generally, *Ascomycota* have higher competitiveness and stress resistance and are important decomposers in the nutrient cycle to improve their dominant position in the soil [25,26]. In our experiment, we also found that the dominant phylum of the soil in the seven organic and inorganic soil management systems was *Ascomycota*. OTUs within the *Ascomycota* were largely identified as members of *Sordariomycetes*, and this proportion was higher in the organic mulches versus inorganic. Similarly, Zhao et al. [27] found an increase in the relative abundance of *Sordariomycetes* in soil treated with *Allium fistulosum* to suppress soil-borne diseases and alleviate ARD in apple orchards. Camacho-Sanchez et al. [28] identified *Sordariomycetes* among the top groups in soils under organic soil management in comparison to conventional soil treatment.

In our study, *Mortierellomycetes* also dominated in the organic systems versus inorganic, which coincides with the observations of Li et al. [29] where organic amendments changed the fungal community composition, with a significant increase in the relative abundances of *Mortierella*. Alpha diversity analysis showed that the greatest fungal diversity occurred in the *Miscanthus*-mulched soil, and it was reduced in mushroom compost in comparison to other mulches.

In this study, we identified fungi species affected by different soil management systems in the orchard. Those which were present exclusively in organic samples included *Mycothermus thermophilus*, *Fusarium venenatum*, *Stropharia rugosoannulata*, *Crassicarpon thermophilum*, *Duddingtonia flagrans*, and unidentified species of *Chaetomiaceae* (*Ascomycota*). *M. thermophilus* belongs to thermophilic fungi which are essential in Phase II of compost formation because they convert nutrients from the raw material into microbial biomass and, in doing so, contribute to the selectivity of mushroom composting [30]. It has been shown that *M*. *thermophilus* (syn. *Scytalidium thermophilum*/*Humicola insolens*) aids the reassimilation of ammonia into compost [31,32,33] and stimulates growth of the button mushroom mycelium; thus, it has the main role in the removal of ammonia and the selectivity of the compost for the growth of *A. bisporus* [34]. In the presence of *M. thermophilus*, the hyphal elongation of *A. bisporus* doubles [35], and fungal competitors of *A. bisporus*, such as *Chaetomium globosum*, are suppressed [31,34]. *M. thermophilus* is the dominant fungal taxon in Phase II compost and makes up most of the microbial biomass in the compost [36,37,38,39,40], but it is just one player in a multifaceted microbial community [30]. *M. thermophilus* is being reported to produce appreciable titers of cellulases and hemicellulases; thus, it has the potential for the hydrolysis of lignocellulosics [41] and is a useful biomarker of compost quality and may be applied as a predictive marker of mushroom crop yields and quality [30]. Similarly, in organic floor management systems, our study distinguished *Crassicarpon thermophilum*, which has been identified as prolific producer of cellobiose dehydrogenase, an important component of the newly discovered oxidative system comprising cellobiose dehydrogenase (CDH) and lytic polysaccharide monooxygenases (LPMOs), which is known for its ability to enhance degradation of lignocellulosics [42].

In this study, we observed that *Fusarium venenatum* specifically occurred in organic mulched soil vs. inorganic. It has been shown that *F. venenatum* can occasionally colonize plant tissues and rarely provokes disease.

The *Stropharia rugosoannulata* exclusively colonizing organic samples was previously found to increase SOM and available phosphorus content on forestland [43]. Moreover, *S. rugosoannulata* is considered the most promising fungal species identified for mycofiltration, as well as one of the most efficient degraders of polycyclic aromatic hydrocarbons among litter-decomposing fungi [44].

It has been shown that *Duddingtonia flagrans* substantially increased the growth and uptake of nutrients of tomato plants (mainly phosphorus—up to 70% in the best treatments). *D. flagrans* is considered to be potentially useful, not just for nematode control but also for promoting plant growth and for increasing nutrient use efficiency [45].

Soil bacteria are widely recognized for their roles in nutrient cycling, such as for nitrogen and phosphorus, and for promoting soil health. In our study, the most prominent phyla, *Proteobacteria*, accounted for 43% of all assigned bacteria OTUs. *Proteobacteria* and *Bacteroidota* were more numerous in organic systems, while *Acidobacteriota* predominated in the inorganic samples. *Gammaproteobacteria*, *Bacteroidia*, and *Alphaproteobacteria* classes were predominant in the organic samples, while *Acidobacteriae*, *Verrucomicrobiae*, and *Gemmatimonadetes* were more abundant in the inorganic mulches. Also Wang et al. [46] found that application of bioorganic fertilizer significantly increased apple yields and shaped bacterial community structure in orchard soil to predominant phyla *Proteobacteria* and *Acidobacteria* and the most dominant classes *Gammaproteobacteria* and *Alphaproteobacteria* in the soil profile [46].Further, Wassermann et al. [47] showed that *Gammaproteobacteria* was the dominating class in apples, followed by *Alphaproteobacteria*, *Actinobacteria*, and *Bacteroidetes*. It has been found that non-nitrifying *Alphaproteobacteria* improve the availability of calcium, magnesium, iron, manganese, and copper [4].

Our study revealed bacteria species that were present in a greater share in organic samples vs. inorganic. One of them was rhizobacterium *Rahnella aquatilis*, especially colonizing *Miscanthus*-mulched soil. Previously identified, its JZ-GX1 strain was demonstrated to potentially induce *A. thaliana* tolerance to iron-deficiency stress by promoting the development of lateral roots and root hairs and increasing the activities of H^+^ ATPase and Fe^3+^ reductase [48]. The use of PGPR to help plants obtain available iron is considered to be environmentally friendly [49]. It is known that several beneficial microorganisms can promote iron uptake by plants based on the mechanisms of chelation, reduction, acidification, and induction, among which the induction of plant systemic resistance mediated by volatile organic compounds (VOCs) has attracted wide attention in recent years [50].

Further, we found more *Pseudomonas brassicaceum* in the organic samples vs. inorganic. *Pseudomonas brassicacearum* and related species of the *P. fluorescens* complex have long been studied as biocontrols and growth-promoting rhizobacteria involved in the suppression of soilborne pathogens [51]. Moreover *P. brassicaceum* has been found to be one of the *Erwinia amylovora* antagonists and is effective for preventive treatment on pear fruits, leading to a necrosis reduction of up to 90% [52]. In previous studies, changes were already observed in rhizosphere microbial communities in apple trees in long-term replanted orchards of central Europe. For instance, *Pseudomonas fluorescens*, *Pseudomonas tolasii*, *Pseudomonas* spp., and *Novosphingobium* spp. were the bacteria which were mainly attributed to gamma-irradiated soils with increased plant growth, while *Fusarium venenatum* was present in native soil [53].

In consistency with the aforementioned findings, we observed a greater share of *Novosphingobium* and *Flavobacterium* in the organic samples. *Novosphingobium*, *Flavobacterium*, and *Pseudomonas* spp. are genera that are considered to be antagonists of soil-borne plant-pathogenic fungi and plant growth-promoting rhizobacteria and have been reported as contributing to a reduced abundance of soil-borne plant pathogens in apple orchards [53].

On the other hand, we found a higher ratio of *Bradyrhizobium* in inorganic floor management systems vs. organic. Well-reported beneficial bacteria *Bradyrhizobium* and *Rubrobacter* were included among the keystone taxa of apples [54]. In the organic samples, we identified more *Sphingomonas*, *Burkholderia*, and WD21-01 soil group than in organic mulches. *Sphingomonas* has been detected as one of the main genera, while *Burkholderia* as the top genus predominated in the apple rhizosphere [55,56]. The unclassified WD21-01 soil group has been determined as a hub of barley and the most frequent and differentially associated node, forming majorly negative associations in host-plant-specific interconnections. It is hypothesized that the WD21-01 soil group was most likely involved with the degradation of plant cell wall components [57]. Moreover, the WD2101 soil group was specifically identified after 10 years of conventional tillage when compared to reduced tillage in almond orchards [58].

## 4. Materials and Methods

**Figure 11:**
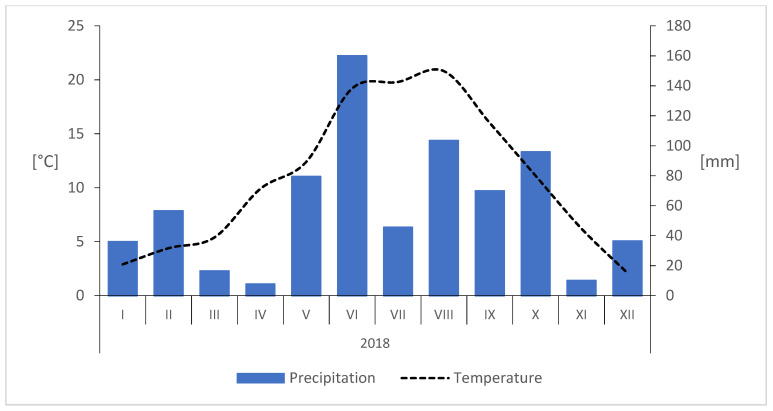
Weather conditions for the experimental site in 2020.

The experimental site was located in an orchard at the Warsaw University of Life Sciences, Wilanów, Poland (N 52°9′36.1″, E 21°5′58.2″). The average annual temperature for the region is 8.6 °C, with the rainfall estimated at 564.5 mm. The weather conditions for the orchard site were monitored using the Davis Vantage Pro 7 field weather station (Davis Instruments, Hayward, CA, USA), which was located near to the experimental plots. The weather data collected during the season of sample collections are presented in Figure 11.

The plant material consisted of the apple *Malus* × *domestica* Borkh. cultivar ‘Red Jonaprince’, with feathered maiden trees grafted on M.9 rootstock. Trees were planted with 3.5 × 1 m spacing in deep, loamy alluvial soil, with 2.5% of humus, and trained in a spindle-bush system.

The experiment was set up in 2017 using a randomized block design. The following methods of soil management in 1 m wide rows of trees were compared: (1) Herbicide strips (HSs) were used as a control, for which a soil width of 1 m was sprayed with herbicide (glyphosate in the Roundup 360 SL formulation at a dose of 4 Lha^−1^) at the beginning of June and after harvesting the fruit at the beginning of October in each year of the experiment; (2) mechanical cultivation (MC), which was used in rows of trees 1 m wide using a rototiller-type tool mounted on the rear of a tractor equipped with a hydraulic system, enabling access to the area between trees, and the soil was tilled up to 10 cm deep up to six times during the growing period (from April to October) depending on weather conditions; (3) synthetic mulch (BC), for which a soil strip 1 m wide was mulched in rows of trees with a black polypropylene-woven ground cover of 100 g per m^2^ density; (4) organic litter (MM), for which a 1 m width of soil was mulched in rows of trees with a 10 cm layer of shredded straw from *Miscanthus* × *giganteus* (75 dm^3^ per tree); (5) organic litter (MMM), for which a soil width of 1 m and depth of 10 cm, before tree planting, was mixed in rows of trees with 75 dm^3^ of shredded straw from *M.* × *giganteus*, and directly after planting soil was mulched in rows of trees with a 10 cm layer of the straw; (6) organic litter (FM), for which a soil width of 1 m was mulched in rows of trees with a 10 cm layer of spent mushroom compost (75 dm^3^ per tree); and (7) organic litter (FMM), for which a soil width of 1 m and depth of 10 cm, before tree planting, was mixed in rows of trees with 75 dm^3^ of spent mushroom compost, and directly after planting soil was mulched in rows of trees with a 10 cm layer of the spent mushroom compost FM. All organic mulches were replenished during the trial to maintain 10 cm layer. The chosen parameters of organic mulches that we used and the physico-chemical soil properties were previously described by Przybyłko et al. [22].

Each combination was replicated three times in plots of five trees, with each plot separated by two protective trees. The same practices of pruning and disease and pest control were applied for all management systems in accordance with the standards of Integrated Pest Management. During the trial, the trees were not fertilized.

Soil samples were collected on the experimental site in August 2020, in the middle of the 4th season of running different floor management systems. Two different specimens per each plot were established, as follows: sample for main physico-chemical properties and rhizosphere soil sample.

A sample containing approximately 1500 g per each experimental plot (each made up of 15 subsamples) was taken for physico-chemical testing using a gouge auger set for stepwise sampling (Eijkelklamp, Giesbeek, The Netherlands). Samples represented 0–40 cm soil layers and were immediately placed at room temperature in the experimental orchard facilities to dry. The measurements were taken under lab conditions. The pH parameters and conductivity were measured in 1-molic KCl solution extract and distilled water extract, respectively, with an Elmetron CPC-505 meter (Elmetron, Zabrze, Poland). For each replication, 10 g samples were used for examination of the macroelement content. Measurements for P, K, and Mg were run using a Thermo Scientific iCAP 6500 Duo spectrometer (Thermo Fischer Scientific, Waltham, MA, USA) using argon with 99% purity as a carrier gas in extracts previously prepared in line with the Egner–Riehm method for P and K and Schachtschabel’s procedure for Mg. Both were previously described in detail by Stafecka and Komosa [59]. Organic matter content was measured according to Tiurin’s method. For that purpose, all plant remnants were removed from the sample. The soil was air dried, grounded with mortar, and sieved with a 0.25 mm sieve. Then a sample of 0.3 g of soil was transferred to 10 mL of a 0.067 M solution of potassium dichromate that was acidified with a sulphuric acid and boiled slowly for 5 min. After cooling down, the solution was titrated with a 0.2 M Mohr’s salt solution to a point when the color started to turn green. On this basis, the organic matter content was calculated, as was described by Łądkiewicz et al. [60].

### 4.1. Sample Collection

Rhizosphere soil samples were collected in August 2020 with a sterile spade, close to the stem and at depths of 10 to 40 cm, where the root system was denser. Soil samples were collected from each combination replicated three times in plots of five trees. All samples were stored in sterile polythene bags and taken the short distance (ca. 7.5 km) back to the WULS laboratory and immediately stored on ice in a cooling storage box for further processing within 24 h of the time of sampling. In accordance with Berlanas et al. [61], the sampled roots with rhizosphere soil particles attached were placed in sterile tubes containing 9 mL of physiological solution (9 g/L NaCl). The tubes were vortexed for 5 min to detach the soil particles and then centrifuged at 4000 rpm for 5 min. The supernatant was discarded, and the remaining soil fraction was stored at −80 °C and used for DNA extraction.

### 4.2. DNA Extraction and Sequencing

The rhizosphere DNA was extracted from a 0.5 g sample of each combination replicated three times using the DNeasy PowerSoil Kit (Qiagen, Hilden, Germany), and replicated DNA samples were pooled prior to sequencing. A metagenomic analysis of the bacterial population was performed based on the hypervariable region V3-V4 of the 16S rRNA gene. The specific sequences of the 341F and 785R primers with adaptors are provided in Supplementary Material Table S1. All steps, including amplification, indexing, and library quantification, were performed according to the 16S Metagenomic Sequencing Library Preparation(Illumina, San Diego, CA, USA) protocol. The PCR reactions were performed with a Q5 Hot Start High-Fidelity 2X Master Mix (New England Biolabs, Ipswich, MA, USA) under the reaction conditions provided in Supplementary Material Table S2. The resulting amplicons were then indexed with a Nextera XT Index Kit (Illumina) using the Q5 Hot Start High-Fidelity 2X Master Mix (New England Biolabs) under the reaction conditions given in Supplementary Material Table S3. The library size was evaluated on a Bioanalyzer 2100 DNA High Sensitivity chip (Agilent). Sequencing was performed on the MiSeq Reagent Kit v3, 2 × 300 PE (paired-end) in order to obtain at least 50,000 read pairs per sample.

A metagenomic analysis of fungal population was performed based on the hypervariable region ITS1 with the specific primers provided in Supplementary Material Table S4. The primers contained an Illumina adaptor sequence (in italics) and an ITS1 locus specific sequence. All further steps (second PCR, library validation, and sequencing) were the same as described above for the V3–V4 16S region.

### 4.3. Bioinformatic Analysis

Automatic preliminary data analysis was carried out on the MiSeq apparatus using the MiSeq Reporter (MSR) v2.6 software. The analysis consisted of two stages: automatic demultiplexing of samples and generation of fastq files containing raw readings. Bioinformatic analysis ensuring the classification of the readings to the species level was carried out with the QIIME software package [62] based on the reference sequences SILVA_v_138 (bacteria) (Quast et al., 2013) and UNITE v8.2 (fungi) [63]. The analysis consisted of the following steps: 1. removal of adapter sequences using the cutadapt program [64]; 2. analysis of the quality of readings and removal of low-quality sequences (quality < 20 (16S) and quality < 30 (ITS), minimum length 30) using the cutadapt program [64]; 3. combination of paired sequences using fastq-join (16S) [65] and SeqPrep (ITS) algorithm; 4. removing sequence chimeras using usearch61 algorithm [66]; 5. clustering based on a selected database of reference sequences using uclust algorithm [66]; and 6. assigning a taxonomy to a selected database of reference sequences using uclust (16S) and BLAST (ITS) algorithms [67].

Further bioinformatics analysis was performed using the R program [68] and using the phyloseq [69], vegan [70], and factoextra packages [71], while the graphs were generated using the ggplot2 [72] and ggbiplot packages [73].

## 5. Conclusions

The results of our study show the microbial constitution of seven floor management regimes. We identified the fungal and bacterial taxonomic units specifically assigned to organic and inorganic regimes, as well as species exclusively attributed to each mulching system. Moreover, the use of spent mushroom substrate contributed to higher phosphorus, potassium, and magnesium, among others, as well as organic matter content, in comparison to other combinations. The results encourage the future exploration of apple rhizosphere microbiomes and their effect on soil regeneration and plant yield and fruit quality.

## Figures and Tables

**Table 1:** Soil properties in fourth growing season after apple orchard establishment.

System	pH [-]	Mineral Element Content in Soil [mg·100 g soil^−1^]			Conductivity [μS·cm^−1^]	Organic Matter Content [%]
		P	K	Mg		
HS	5.22 a *	1.23 a	12.4 a	15.5 a	135 a	1.58 a
MC	5.68 a	0.89 a	12.0 a	15.3 a	112 a	1.53 a
BC	5.20 a	1.08 a	12.2 a	15.0 a	126 a	1.45 a
MM	5.30 a	1.27 a	14.2 a	16.0 a	117 a	1.58 a
MMM	5.30 a	0.98 a	14.0 a	16.9 a	135 a	1.80 a
FM	5.92 ab	15.0 a	73.3 b	18.9 ab	942 b	2.96 b
FMM	6.40 b	14.9 b	74.6 b	21.2 b	982 b	2.96 b

* means followed by the same letter within column and do not differ significantly at *p* ≤ 0.05. Abbreviations: HS—herbicide strip, MC—mechanical cultivation, BC—synthetic mulch, MM—*Miscanthus* mulch, MMM—*Miscanthus* mulch mixed with the soil, FM—mushroom compost, FMM—mushroom compost mixed with the soil.

**Table 2:** Alpha diversity indices of fungal and bacterial amplicon sequencing data depending on the soil maintenance system. Abbreviations: HS—herbicide strip, MC—mechanical cultivation, BC—synthetic mulch, MM—*Miscanthus* mulch, MMM—*Miscanthus* mulch mixed with the soil, FM—mushroom compost, FMM—mushroom compost mixed with the soil.

Sample	Fungal Indices				Bacterial Indices			
	Shannon	Simpson	Chao1	ACE	Shannon	Simpson	Chao1	ACE
HS	5.66	0.98	2624	2604	7.47	1.00	6121	6044
MC	5.33	0.98	2582	2548	7.48	1.00	5926	5933
BC	5.34	0.98	2559	2518	7.48	1.00	6147	6000
MM	6.14	0.99	3011	2986	7.45	1.00	7422	7472
MMM	6.15	0.99	2747	2726	7.41	1.00	6216	6158
FM	4.91	0.96	1939	1920	6.71	0.99	4707	4525
FMM	3.21	0.76	1746	1713	6.99	1.00	5088	4800

## Data Availability

Not applicable.

## Supplementary Materials

The following supporting information can be downloaded at: https://www.mdpi.com/article/10.3390/ijms24129898/s1.
