# Supplementary material for: Tree Root-Associated Microbial Communities Depend on Various Floor Management Systems in an Intensive Apple (Malus × domestica Borkh.) Orchard
Source: Int J Mol Sci. 2023 Jun 8;24(12):9898. doi: 10.3390/ijms24129898 (PMC10297936; doi:10.3390/ijms24129898)
Supplement: Supplementary file 1 [file ijms-24-09898-s001.zip › ijms-2372068-supplementary.pdf]

Supplementary Material

Table S1. The specific sequences of the 341F and 785R primers with adaptors. The primers contain Illumina adaptor sequence (in italics) and V3-V4 16S rRNA locus specific sequence.

Primer	Sequence
341F	5′ *TCGTCGGCAGCGTCAGATGTGTATAAGAGACAG*CCTACGGGNGGCWGCAG
785R	5′ *GTCTCGTGGGCTCGGAGATGTGTATAAGAGACAG*GACTACHVGGGTATCTAATCC

Table S2. PCR conditions for analysis of bacterial and fungal population.

Steps	Temperature (°C)	Duration	Cycles
Initial denaturation	98	30 seconds	1
Denaturation	98	10 seconds	25
Annealing	55	30 seconds	
Extension	72	20 seconds	
Final denaturation	72	2 minutes	1

Table S3. PCR conditions for amplicon indexing.

Steps	Temperature (°C)	Duration	Cycles
Initial denaturation	98	30 seconds	1
Denaturation	98	10 seconds	7
Annealing	65	30 seconds	
Extension	72	20 seconds	
Final denaturation	72	2 minutes	1

Table S4. The specific sequences of the ITS1FI2 and 8S primers with adaptors. The primers contain Illumina adaptor sequence (in italics) and ITS1 locus specific sequence.

Primer	Sequence
ITS1FI2	5′ *TCGTCGGCAGCGTCAGATGTGTATAAGAGACAG*GAACCWGCGGARGGATCA
5.8S	5′ *GTCTCGTGGGCTCGGAGATGTGTATAAGAGACAG*CGCTGCGTTCTTCATCG
